# Reduced efficacy of an anti-toxin vaccine from senescence-driven attenuation of toxin virulence

**DOI:** 10.1172/jci.insight.199834

**Published:** 2026-06-08

**Authors:** Xin Du, Ching Wen Tseng, Elisabet Bjånes, Hunter Gage, Jaclyn Swan, Chih-Ming Tsai, Irshad A. Hajam, Cesia Gonzalez, Brian Lin, Victor Nizet, George Y. Liu

**Affiliations:** 1 Department of Pediatrics, UCSD, San Diego, California, USA.; 2 Division of Infectious Diseases, Rady Children’s Hospital San Diego, San Diego, California, USA.; 3 Orca Bio, Menlo Park, California, USA.; 4 Department of Pharmacy, UCSD, San Diego, California, USA.; 5 Department of Ecological Plant and Animal Sciences, La Trobe University, Melbourne, Victoria, Australia.

**Keywords:** Infectious disease, Microbiology, Vaccines

## Abstract

It remains unclear why vaccines targeting prominent microbial virulence factors often fail in clinical trials. Because microbial virulence depends on interaction with the host immune system, we investigated how changes in host immune function alter vaccine efficacy. Using a vaccine against *Staphylococcus aureus* α-toxin (Hla), which targets host metalloprotease ADAM10 on myeloid cells, we show that Hla virulence is reduced in aged mice due to diminished ADAM10 activity and impaired myeloid cell function. Depletion of myeloid cells with cyclophosphamide in young mice similarly reduced toxin virulence. Immunization against Hla conferred strong protection against *S*. *aureus* infection in young but not aged mice. These findings indicate that pathogenic functions of microbial factors characterized in immunocompetent young animals may not predict virulence or vaccine efficacy in immunocompromised hosts. These findings underscore the need to account for host immune status in the development and evaluation of vaccines targeting microbial virulence factors.

## Abstract 2

The efficacy of vaccines against staphylococcal alpha toxin is markedly reduced in aged mice because of senescence-related loss of toxin virulence function.

## Introduction

Studies of host-pathogen interactions provide the fundamental basis for understanding how microbes establish infection and inflict damage in host tissues. Prominent virulence factors unveiled from these studies serve as targets for translational intervention (1–3), providing a rationale for sustained public support of host-pathogen research (4). Microbial virulence is most investigated in young adult laboratory rodents at the peak of immune competence. Yet infectious pathology arises from dynamic interactions between microbial factors and host immune components, including activation of downstream effectors and evasion of immune defenses (3). Thus, the manifestation of microbial virulence depends on an optimally functioning host immune system.

However, immune functions vary substantially across the human lifespan and with clinical conditions such as prematurity, aging, diabetes, or immunosuppressive drug use. These immune-altering states may shift the balance of host-pathogen interactions and thereby affect the expression or impact of microbial virulence. We hypothesized that virulence defined in young adult animals may not translate directly to immunocompromised hosts and that this discrepancy could contribute to the failure of vaccines that target virulence factors.

To test this hypothesis, we focused on α-toxin (Hla), a major *Staphylococcus aureus* virulence determinant that has been extensively studied in young rodents (5, 6). Hla exerts its pathogenic effects through ligation of the host metalloprotease ADAM10, leading to cellular damage, skin barrier disruption, and exacerbated inflammation (7). Upon binding ADAM10, Hla triggers cleavage of junctional and adhesion molecules and activates proinflammatory signaling cascades that amplify tissue injury (7–9). Hla contributes to the pathogenesis of multiple *S*. *aureus* infections (5, 6), which collectively accounted for over one million deaths worldwide in 2019 (10).

Despite decades of research and more than 10 phase II or III clinical trials, there is still no licensed vaccine against *S*. *aureus* to date (11, 12). Various explanations have been proposed, including poor immunogenicity, suboptimal adjuvants, and inappropriate trial design that enrolled patients with impactful comorbidities (13). A particularly relevant example is the trial of suvratoxumab, a human monoclonal antibody targeting Hla, which failed to meet its primary endpoints in preventing *S*. *aureus* pneumonia (14). Notably, a subgroup analysis showed that suvratoxumab was effective in patients under 65 years of age but conferred no benefit in those over 65 (14). This trial outcome raises the critical question of whether age-associated changes in immune function alter the pathogenic potential of virulence factors like Hla. In this study, we tested whether Hla-mediated pathology is reduced in aged or immunosuppressed hosts and whether such changes influence vaccine efficacy. Our findings have implications for understanding why some virulence-targeting interventions succeed in preclinical models yet fail in real-world clinical settings.

## Results

### Evaluation of Hla as a virulence factor and vaccine target in aged mice.

**Figure 1:**
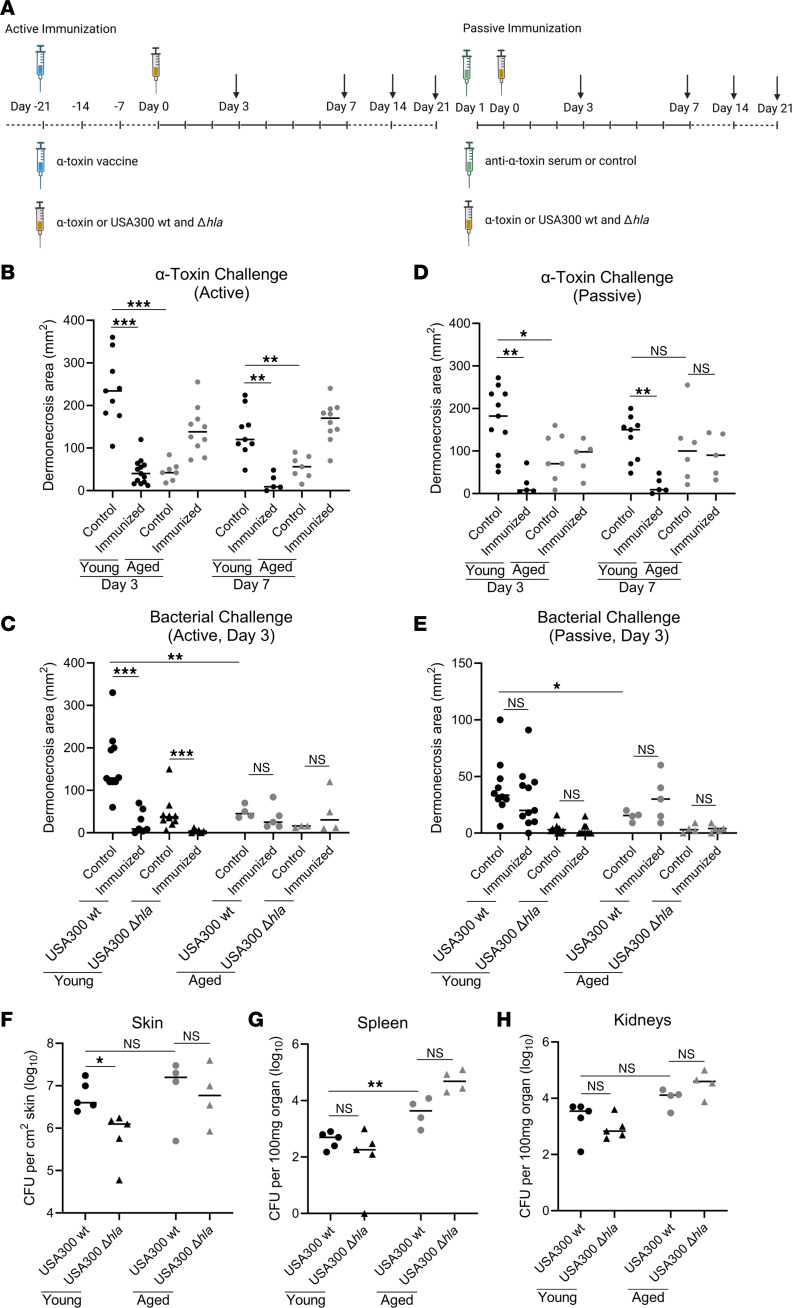
Evaluation of Hla as a virulence factor and vaccine target in aged mice. (**A**) Schematic: Young and aged mice were immunized subcutaneously (s.c.) against Hla (left) or administered intraperitoneally mouse anti-Hla serum from Hla toxoid–vaccinated mice (right), then challenged s.c. with Hla, WT or Δ*hla* *S*. *aureus* (USA300). (**B**) Active immunization: Skin lesion sizes 3 and 7 days after Hla challenge (*n* = 5–9 mice). (**C**) Passive immunization: Skin lesion sizes 3 and 7 days after Hla challenge (*n* = 5–9). (**D**) Active immunization: Skin lesion sizes 3 days after WT or Δ*hla* *S*. *aureus* (USA300) subcutaneous infection (*n* = 9–11 young, 4–7 aged). (**E**) Passive immunization: Skin lesion sizes 3 days after WT or Δ*hla* *S*. *aureus* (USA300) subcutaneous infection (*n* = 9–11 young, 4–7 aged). (**F**–**H**) Bacterial burden in the skin, spleen, and kidneys of previously unimmunized young and aged mice, 3 days after *S*. *aureus* challenge (*n* = 5 young mice, 4 aged mice). Line in **A**–**H** represents the median. *P < 0.05; **P < 0.01; ***P < 0.001 by Kruskal-Wallis non-parametric 1-way ANOVA test (**B**–**H**). NS, not significant; USA300 WT, *S*. *aureus* USA300 WT strain SF8300; USA300 Δ*hla*, *S*. *aureus* USA300 SF8300 Hla isogenic mutant. Each data point represents an individual mouse. Arrows at days 3, 7, 14, and 21 denote the time when skin lesions were assessed. The schematic in **A** was created in BioRender (https://BioRender.com/49kavfj, https://BioRender.com/nqcey3h).

To assess whether Hla virulence is impaired with old age, we performed subcutaneous challenge of young and aged mice with Hla toxin or a wild-type (WT) USA300 or an isogenic Hla deletion mutant (Δ*hla*) (Figure 1A). For assessment of anti-Hla vaccine efficacy, some groups of mice also received either active immunization (α-toxoid plus alum) or passive transfer of protective sera; “young” refers to mice 6–12 weeks of age and “aged” refers to mice aged 16 to 22 months.

In vivo, subcutaneous injection of Hla induced markedly smaller dermonecrotic lesions in aged mice than in young mice (Figure 1, B and C, and Supplemental Figure 1, A and B; supplemental material available online with this article; https://doi.org/10.1172/jci.insight.199834DS1). Subcutaneous challenge with WT or Δ*hla* *S*. *aureus* corroborated these findings; over a 21-day infection, Hla contributed to larger lesion sizes and greater skin bacterial burden in young mice but had a modest effect in aged mice (Figure 1, D and E, and Supplemental Figure 1, C–H). Notably, *S*. *aureus* dissemination to deep tissues was greater in aged mice and was independent of Hla activity (Figure 1, F–H, and Supplemental Figure 1, I and J). These findings provide direct evidence that Hla virulence is attenuated in aged hosts,

In line with published Hla vaccine studies (6, 15), active immunization significantly reduced lesion sizes following challenge with either Hla (Figure 1B and Supplemental Figure 1A) or *S*. *aureus* in young mice (Figure 1D and Supplemental Figure 1, C, E, and G). In contrast, aged mice showed reduced or no protection under the same vaccination regimen. Active Hla vaccination of aged mice followed by Hla challenge, unexpectedly, aggravated the severity of skin lesions for unclear reason (Figure 1B).

For passive immunization studies, sera from vaccinated young mice were adoptively transferring into young or aged recipients prior to toxin or *S*. *aureus* challenge (Figure 1, C and E, and Supplemental Figure 1, B, D, F, and H). The sera protected young mice against necrotic lesions after subcutaneous Hla challenge, but unexpectedly conferred no significant protection to young mice except on day 14 after infection, contrary to well-established efficacy of anti-Hla antibody therapy against Hla-mediated pathology (16, 17). We speculate that the finding likely results from the use of sera with reduced antibody activity. Irrespectively, our collective results suggest that the preserved pathogenic function of the microbial target in the host (Hla) is an important determinant of the success of the vaccine.

### Reduced ADAM10 activity contributes to decreased Hla virulence in senescent mice.

**Figure 2:**
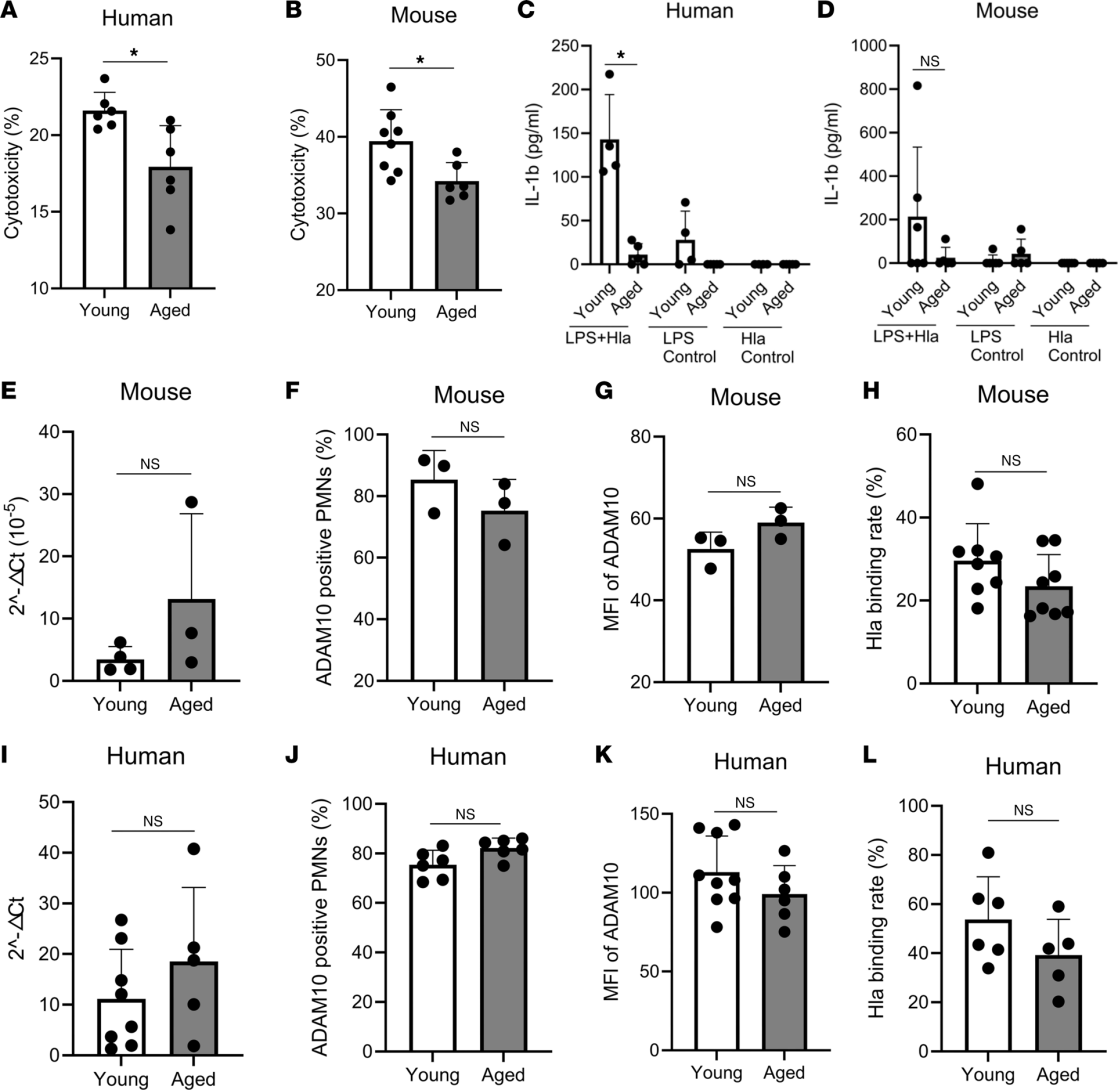
Hla activity and ADAM10 expression in aged hosts. (**A**) Hla lysis of human neutrophils assessed using LDH assay. Percentage cell lysis in baseline untreated control: young 13.8, aged 14.6 (*n* = 6 humans). 10 U/mL Hla was used for all in vitro experiments. (**B**) Hla lysis of mouse neutrophils assessed using LDH assay. Percentage cell lysis in untreated control: young 12.0, aged 11.1 (*n* = 6–8 mice). (**C**) IL-1β release by LPS-primed human neutrophils after Hla stimulation (*n* = 4–5 humans). (**D**) IL-1β release by LPS-primed murine neutrophils after Hla stimulation (*n* = 5–6 mice). (**E**) ADAM10 expression by neutrophils from young and aged mice measured by qPCR (*n* = 3–4 mice). (**F** and **G**) Percentage and mean fluorescence intensity (MFI) of ADAM10 expression on murine neutrophils, as assessed by flow cytometry (*n* = 3). (**H**) Binding of Hla to murine neutrophils assessed by flow cytometry (*n* = 7–8). (**I**) ADAM10 expression by neutrophils from young and aged humans measured by qPCR (*n* = 5–8). (**J** and **K**) Percentage and MFI of ADAM10 expression on human neutrophils, as assessed by flow cytometry (*n* = 6). (**L**) Binding of Hla to human neutrophils assessed by flow cytometry (*n* = 5–6). Each data point represents an individual mouse or human. The data in **A**–**L** are presented as mean ± SEM of biological replicates. *P < 0.05 by 2-tailed non-parametric Mann-Whitney *U* test (**A**–**D**). NS, not significant.

To investigate the mechanism underlying the reduced virulence of Hla in aged host, we studied 2 well-characterized pathologic functions of Hla — its cytolytic and proinflammatory properties. We incubated neutrophils purified from young and aged mice or human donors with Hla. For humans, “young” refers to volunteers aged 18–35 years and “aged” refers to volunteers aged 65–85 years. Neutrophils from aged humans and mice were more resistant to cytolysis than those from young donors (Figure 2, A and B). IL-1β release was also lower in aged hosts; while differences in mouse IL-1β were modest and did not reach statistical significance, the trend was consistent with human data (Figure 2, C and D). Notably, challenge of neutrophils with Hla alone was insufficient to induce detectable release of active IL-1β. Thus, we primed the neutrophils with LPS, an activator of NF-κB, to elicit pro-IL-1β, and then added the Hla to activate NLRP3 to convert pro-IL-1β protein to its mature form. Hla mediates various pathologic functions through ligation of ADAM10 on myeloid cells, including neutrophils (5). We therefore questioned whether ADAM10 expression or activity differs in neutrophils derived from young and aged mice and humans. ADAM10 transcript and protein levels, as measured by quantitative PCR (qPCR) and flow cytometry, showed no significant differences between groups (Figure 2, E–G and I–K, and Supplemental Figures 2 and 3). Consistent with these findings, Hla binding to neutrophils from young and aged mice and humans was equivalent (Figure 2, H and L, and Supplemental Figure 4). We also evaluated the effect of Hla on another major target of the toxin, macrophages, derived from young and aged mice (18). In the presence or absence of Hla, BMDMs from young and aged mice expressed equivalent levels of ADAM10, with the exception that surface expression of ADAM10 was higher in aged macrophages in the absence of Hla stimulation (Supplemental Figure 5, A–C). Unlike neutrophils, young and aged BMDMs were equally susceptible to Hla cytolysis (Supplemental Figure 5D) and release similar levels of proinflammatory cytokines in response to Hla (Supplemental Figure 5, E–H).

**Figure 3:**
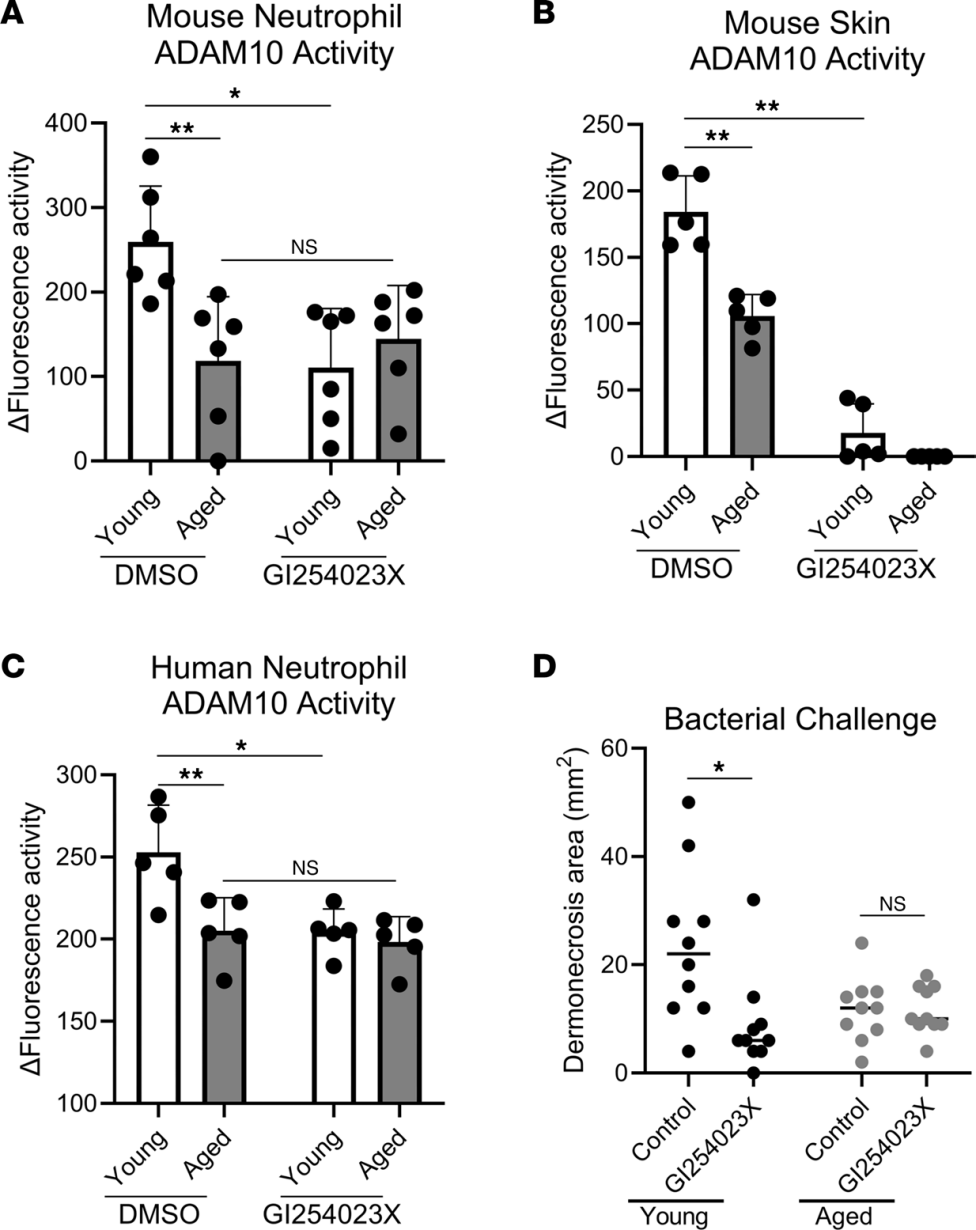
Reduced ADAM10 activity and contribution to diminished Hla activity in aged hosts. (**A** and **B**) Enzymatic activity of ADAM10 from murine neutrophil and skin lysates measured with fluorescence-conjugated substrate (*n* = 5–6). (**C**) Enzymatic activity of ADAM10 from human neutrophil lysates, measured with fluorescence-conjugated substrate (*n* = 5). (**D**) Skin lesion sizes in young and aged mice treated with ADAM10 inhibitor GI254023 followed by infection with *S*. *aureus* (*n* = 10). Each data point represents an individual human volunteer (**A**–**D**). The data are presented as mean ± SEM of biological replicates. Line in **D** represents the median. *P < 0.05; **P < 0.01 by Kruskal-Wallis non-parametric 1-way ANOVA test (**A**–**D**). NS, not significant; GI254023X, ADAM10 inhibitor in DMSO; control, DMSO.

A previous study in Alzheimer disease noted that decreased ADAM10 activity contributed to disease pathogenesis (19), prompting us to evaluate ADAM10 enzymatic function in neutrophils, macrophages, and skin tissues. These analyses revealed reduced ADAM10 activity in both myeloid cell lineages and skin from aged mice compared with young mice (Figure 3, A and B, and Supplemental Figure 5I), reflecting diminished activity in myeloid and likely skin parenchymal cells, including known Hla target, keratinocytes (20). ADAM10 enzymatic activity was also lower in neutrophils from elderly adults, compared with those from young adults (Figure 3C). To investigate the contribution of altered ADAM10 activity to Hla virulence, we treated mice with the ADAM10 inhibitor GI254023 in the context of *S*. *aureus* subcutaneous infection. Inhibition of ADAM10 markedly reduced lesion sizes in young mice, but had no impact in aged mice, consistent with a functional role for ADAM10 in toxin-mediated pathology only in the young group (Figure 3D and Supplemental Figure 6). It is notable that dermonecrotic lesions induced by *S*. *aureus* in Figure 3D are smaller than those similarly induced in Figure 1. DMSO, which was used as a vehicle control in the experiment, can reduce skin lesion sizes when administered with select drugs (21, 22). Irrespective, our findings are consistent with the interpretation (a) that age is associated with altered ADAM10 and Hla activity in myeloid cells, and (b) that these findings are consistent with a contribution of Hla to age-dependent disease outcomes, at least partially through reduced ADAM10 activity in myeloid cells.

### Hla virulence is attenuated in NSG mice engrafted with bone marrow from aged mice.

**Figure 4:**
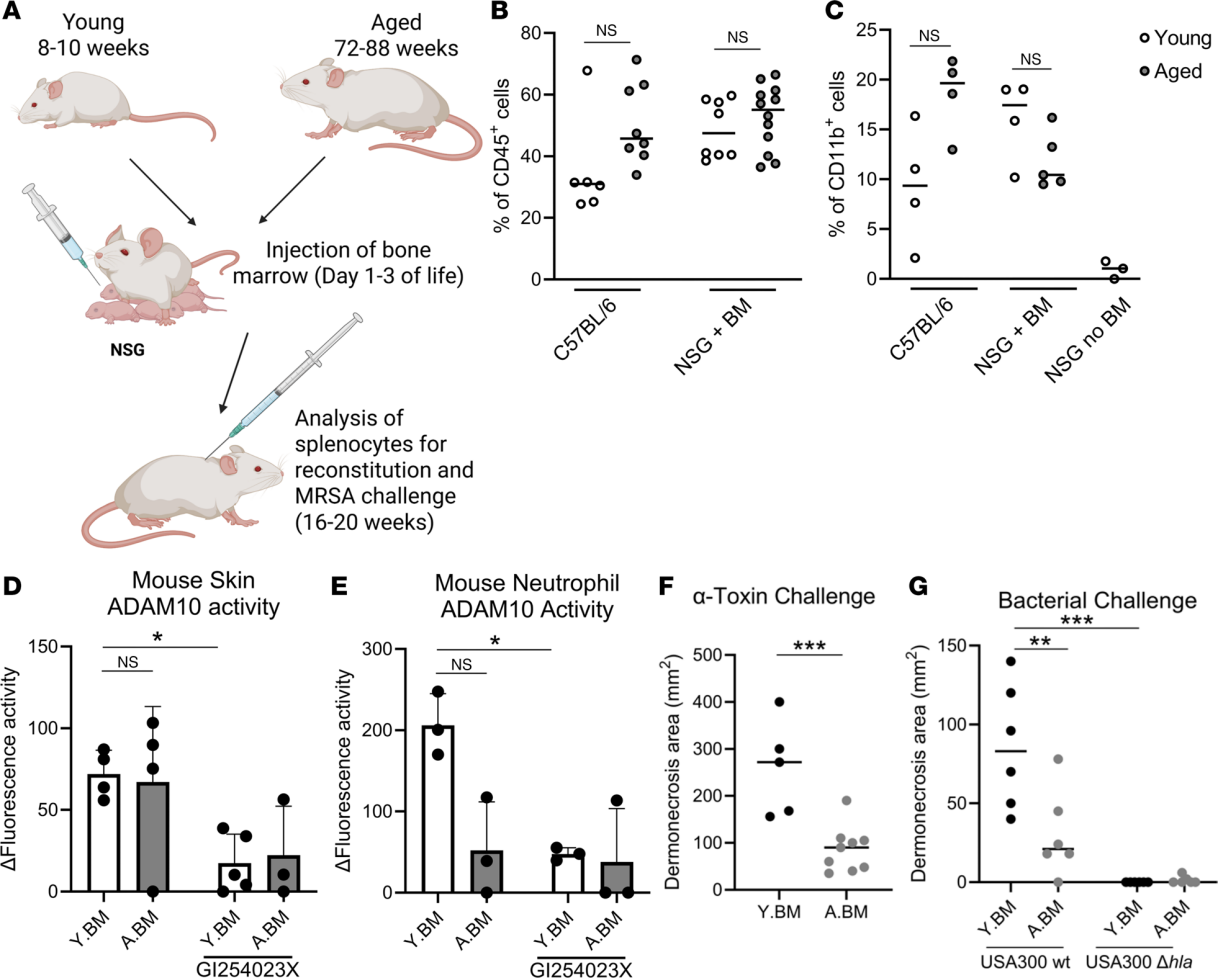
Bone marrow–derived immunocytes contribute to reduced Hla activity in aged mice. (**A**) Schematic: Bone marrow cells from young or aged mice were injected into 1- to 3-day-old NSG mice. At 16–20 weeks of age, the mice were analyzed for reconstitution and then challenged with Hla or *S*. *aureus*. (**B** and **C**) Percentage of splenic CD45^+^ cells and blood CD11b^+^ cells (*n* = 6–12 for CD45 and *n* = 3–4 for CD11b analysis). (**D** and **E**) Enzymatic activities of ADAM10 in neutrophil and skin lysates (*n* = 3–4). (**F**) Skin lesion sizes 3 days after Hla challenge (*n* = 5–9). (**G**) Skin lesions sizes 3 days after infection with WT or *Δhla S*. *aureus* (*n* = 5–6). Each data point represents an individual mouse. Line in **B**, **C**, **F**, and **G** represents the mean. The data in **D** and **E** are presented as mean ± SEM of biological replicates. *P < 0.05; **P < 0.01; ***P < 0.001 by 2-tailed non-parametric Mann-Whitney *U* test (**B**, **C**, and **F**) or Kruskal-Wallis non-parametric 1-way ANOVA test (**D**, **E**, and **G**). NS, not significant; NSG, NOD/SCID/IL2rγ^null^ mouse; Y.BM, NSG recipients of bone marrow from young C57BL/6 mice; A.BM, NSG recipients of bone marrow from aged C57BL/6 mice; GI254023X, ADAM10 inhibitor in DMSO; control, DMSO. The schematic in **A** was created in BioRender (https://BioRender.com/h2vi3i1).

To assess the relative contribution of myeloid cells to Hla activity in young and aged mice, we performed bone marrow transplantation using donors of different ages (Figure 4A). Bone marrow from young or aged mice was transplanted into newborn NOD/SCID/IL2Rγ^null^ (NSG) mice, which lack functional innate and adaptive immune systems (23). At 16 weeks after engraftment, recipients of young and aged marrow exhibited equivalent reconstitution of hematopoietic compartments, as evidenced by comparable frequencies of CD45^+^ cells in the spleen and CD11b^+^ myeloid cells in the blood (Figure 4, B and C). In contrast with cutaneous tissues from naive young and aged mice (Figure 3B), ADAM10 activity in whole skin was similar between the 2 NSG recipient groups (Figure 4D), suggesting that ADAM10 function in non-hematopoietic parenchymal tissues (e.g., keratinocytes) was not affected by the age of the donor marrow. However, neutrophils from recipients of aged marrow exhibited reduced ADAM10 activity compared with recipients of young marrow (Figure 4E), which aligns with findings in intact aged mice (Figure 3A). When challenged with subcutaneous Hla, NSG mice engrafted with aged marrow developed smaller lesions than those receiving young marrow (Figure 4F and Supplemental Figure 7, A and B). This finding was further validated in infections with WT and Δ*hla* *S*. *aureus* strains (Figure 4G and Supplemental Figure 7, C and D). Together, these findings demonstrate that hematopoietic cells, particularly ADAM10-expressing myeloid cells, are likely important contributors to mediating Hla pathogenesis, and that age-associated reductions in their function are sufficient to attenuate toxin virulence.

### Drug-induced leukopenia limits Hla-mediated pathology.

**Figure 5:**
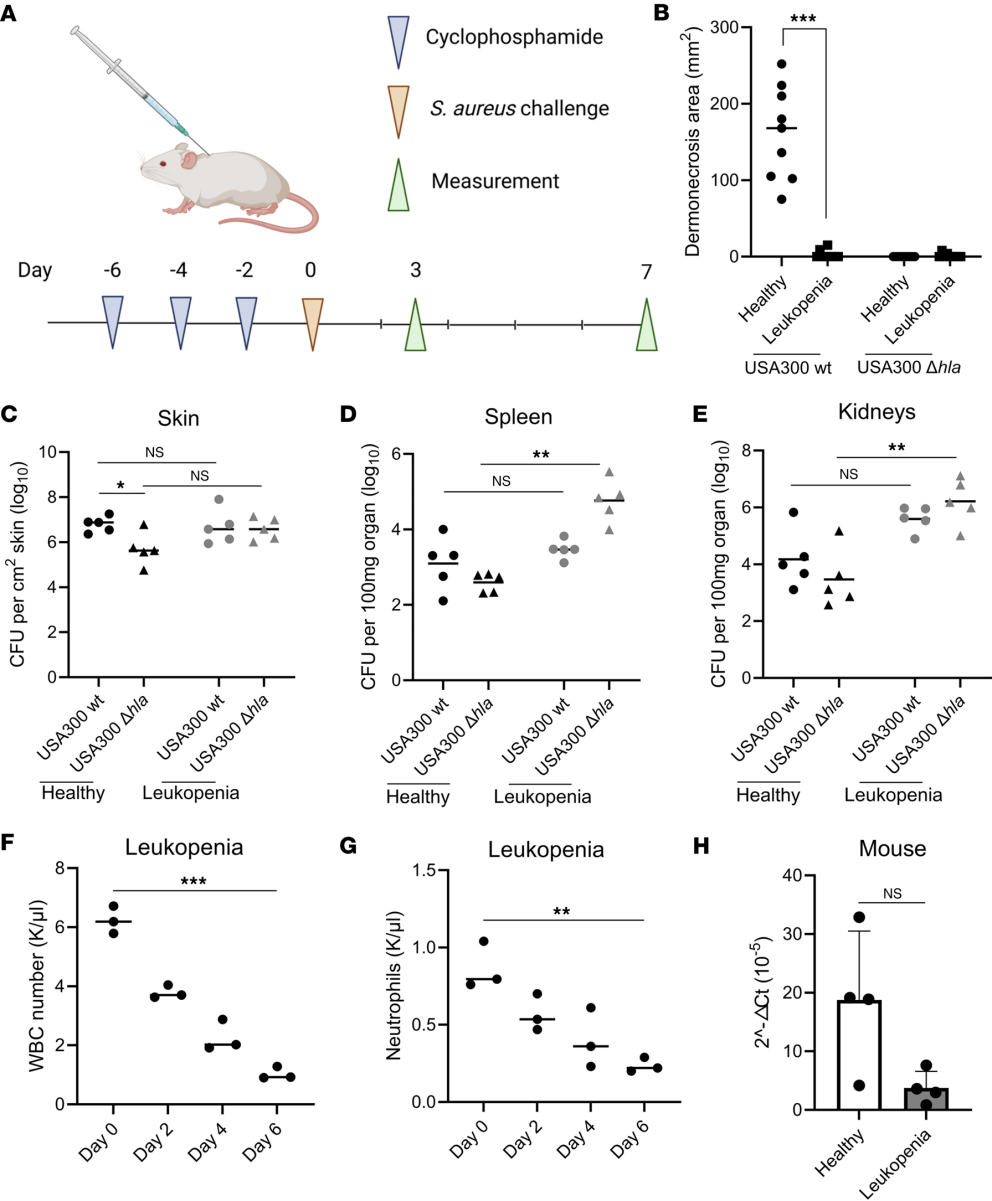
Reduced Hla virulence in mice moderately immunosuppressed with cyclophosphamide. (**A**) Schematic: Mice were administered moderate doses of cyclophosphamide (75 mg/kg), then challenged with WT or *Δhla* *S*. *aureus*. (**B**) Skin lesion sizes 3 days after *S*. *aureus* challenge (*n* = 9). (**C**) Skin bacterial burden 3 days after *S*. *aureus* challenge (*n* = 5). (**D** and **E**) Spleen and kidney bacterial burden 3 days after *S*. *aureus* challenge (*n* = 5). (**F** and **G**) White blood cell and neutrophil cell count after cyclophosphamide treatment (*n* = 3 mice). (**H**) Neutrophil ADAM10 expression quantified by qPCR (*n* = 4 mice). Each data point in **B**–**H** represents an individual mouse. The data in **H** are presented as mean ± SEM of biological replicates. Line in **B**–**G** represents the mean. *P < 0.05; **P < 0.01; ***P < 0.001 by 2-tailed non-parametric Mann-Whitney *U* test (**H**) or Kruskal-Wallis non-parametric 1-way ANOVA test (**B**–**G**). NS, not significant. The schematic in **A** was created in BioRender (https://BioRender.com/pxqt2wr).

In addition to senescence, we hypothesized that depletion or dysfunction of myeloid cells, such as that induced by chemotherapeutic treatment, would similarly diminish Hla virulence. To test this, we employed a well-characterized murine model of leukopenia using serial moderate-dose cyclophosphamide injections (Figure 5A). In this model, WT mice infected subcutaneously with either WT or Δ*hla* *S*. *aureus* developed similar lesion sizes and skin bacterial burdens (Figure 5, B and C), in contrast with the Hla-dependent pathology observed in immunocompetent controls. In deep tissue sites, cyclophosphamide-treated mice exhibited increased bacterial burden that was not dependent on Hla (Figure 5, D and E).

Complete mouse blood count confirmed that cyclophosphamide treatment induced sustained reductions in circulating white blood cells and neutrophils across multiple time points (Figure 5, F and G). Neutrophils from drug-treated animals did not exhibit a significant change in ADAM10 mRNA level, as measured by qPCR (Figure 5H). These findings corroborate the reduced pathogenic function of Hla in a different model of human immunodeficiency, where alternative immune mediators of Hla activity are depleted.

## Discussion

This current study builds on insights from the suvratoxumab trial (14) to test the hypothesis that the virulence of a major *S*. *aureus* toxin, Hla, is diminished in aged or immunocompromised hosts, thereby reducing vaccine efficacy. We show that Hla exhibits age-related attenuation of its cytolytic and proinflammatory function in both murine and human neutrophils. Mechanistically, we find that this reduced pathogenicity is (at least partly) linked to the diminished activity, but not expression, of its host receptor, ADAM10. We find similar reduction of ADAM10 activity in BMDMs derived from aged mice. This loss of ADAM10 function in myeloid cells (and potentially in lymphoid cells) impairs the toxin’s ability to damage tissue and incite inflammation in vivo. Bone marrow chimera experiments further implicate myeloid cells (and potentially lymphoid cells) as key contributors to this shift in Hla activity. In both active and passive immunization models, diminished Hla function translated to loss of vaccine efficacy in aged hosts, even as the overall severity of *S*. *aureus* infection is increased, as reflected by bacterial dissemination.

These findings add to existing hypotheses for the repeated failure of *S*. *aureus* vaccines, including ineffective antigen or adjuvant selection, flawed trial designs, or inappropriate target populations (13). Additionally, we previously showed that preexisting anti–*S*. *aureus* antibodies could compete with suvratoxumab for binding, thereby reducing its efficacy in mice (24). Here we add another explanation: that diminished virulence of microbial antigen targets in aged or immunocompromised individuals may itself reduce vaccine effectiveness. This concept aligns with the suvratoxumab trial outcome, where protection was evident in individuals under 65 but not in those older than 65 (14).

A second important point is that microbial virulence is not intrinsic to the pathogen alone, as it reflects an interaction with the host immune system. Direct engagement of host immune receptors and downstream effectors by microbial factors is typically required to induce tissue damage or to evade immune responses. When these host receptors or effectors are altered, due to aging, disease, or immunosuppression, virulence factors could lose their pathogenic potential. Consistent with this hypothesis, perturbation of effector functions downstream of the Hla receptor ADAM10, such as neutrophil depletion or impaired neutrophil function caused by moderate-dose cyclophosphamide, was sufficient to reduce Hla-mediated pathology. Based on these findings, we propose that other forms of human immunodeficiency, including diabetes, autoimmune or rheumatologic disease, and therapeutic immunosuppression, may similarly alter the function and clinical impact of microbial virulence factors.

The proposition raises a larger question: Is there wider evidence that virulence factor activity is modulated by host immune status? A previous screen by Missiakas and colleagues tested 8 *S*. *aureus* mutants, including Hla and multiple adhesins, in a model of drug-induced severe leukopenia (25). In that systemic infection model, 7 of 8 mutants showed no attenuation in virulence (25), suggesting that their pathogenic function was dispensable in immunocompromised hosts. Similarly, our prior work demonstrated that *S*. *aureus* nuclease lost immune evasion function in aged mice because of reduced neutrophil extracellular trap (NET) formation (26). While neither of these studies raised the broader hypothesis across immunocompromised hosts, together they support the hypothesis that virulence factor functions would be frequently altered in immunocompromised settings. These findings challenge current vaccine development strategies, which often rely on virulence factors defined in young immunocompetent animals and do not account for the immune status of target human populations.

Our study has several limitations. Most experiments were conducted in animal models, and although we confirmed the reduced Hla activity and diminished Hla responsiveness in neutrophils from aged human donors, the relevance to human disease remains to be fully validated. We also evaluated the pathogenic role of Hla in a skin infection model, whereas suvratoxumab was assessed in a human respiratory infection trial. Notably, Hla plays a critical role in both skin and lung infections (27, 28). Thus, we speculate that reduced Hla activity would have a similarly profound effect on host responses in both skin and lung infections. We did not evaluate whether microbial gene expression is altered in aged or immunocompromised hosts, although consistent findings across both purified toxin and live bacterial challenge suggest that altered toxin function has a principal role in the observed phenotypes.

Although diminished Hla function likely contributes to Hla vaccine failure, other factors may play a role, including impaired adaptive immune responses or reduced synergy between transferred antibodies and innate immune effectors in aged hosts (29). Although we established the importance of ADAM10 activity and bone marrow–derived myeloid (and possibly lymphoid) cells in mediating changes in Hla virulence, we did not dissect all downstream effector pathways. Hla is known to induce NALP3 inflammasome activation and mature IL-1β secretion (30), and ADAM10 acts as a sheddase that releases various cytokines to promote inflammation (31). These downstream immune responses may be blunted or altered in aged hosts and warrant further study, although they do not affect the primary conclusion of this work. We also acknowledge that age-associated myeloid dysfunctions beyond ADAM10 could contribute to attenuating Hla-mediated damage. For example, aging has been linked to reduced NETosis and impaired neutrophil chemotaxis, which could independently limit dermonecrosis and inflammation (26, 32, 33).

For the cyclophosphamide study, it is notable that dermonecrotic lesions that develop following cyclophosphamide and *S*. *aureus* treatment are smaller compared with *S*. *aureus* treatment alone, given prior studies that demonstrate increased dermonecrosis with bacterial infections following neutrophil depletion (34). Various host cells, including macrophages, neutrophils, and keratinocytes, play critical roles in the development of dermonecrosis during infections (20, 35, 36). Neutrophil depletion allows *S*. *aureus* to proliferate and to promote dermonecrosis presumably through amplified bacterial factors acting on non-neutrophilic immune cells (34). In our current model, a lower-dose cyclophosphamide regimen partially depletes both neutrophils and other myeloid cells, unlike neutrophil-specific depleting antibodies. Under this condition, we did not see an increase in bacterial burden when the necrotic lesions were measured. Thus, we speculate that the reduced dermonecrosis noted in the mice treated with cyclophosphamide results from (a) decrease in the number (and activity) of myeloid cells that drive dermonecrosis upon Hla activation compared with mice depleted of neutrophils by antibodies, and (b) the absent increase in bacterial number and toxins that drive dermonecrosis compared with mice treated with neutrophil-depleting antibodies.

We observed unexpected exacerbation of skin lesions in aged mice that have been Hla vaccinated and then challenged with Hla but not *S*. *aureus*. Although we did not investigate the mechanism, aged hosts demonstrate greater overall Th17 responses that have been linked to immunopathology, compared with young mice (37, 38). Therefore, it is conceivable that Th17 responses are beneficial for the clearance of live *S*. *aureus*, whereas Th17 responses could induce greater immunopathology when only Hla toxin is used for challenge.

In summary, our findings reveal an apparent paradox: diminished virulence of a major staphylococcal toxin in hosts who are more vulnerable to bacterial infection overall. Immunocompromised patients frequently receive antimicrobial prophylaxis to reduce the risk of severe infection, and vaccination remains a prevention strategy in those who can mount an effective immune response (39). In elderly adults, strategies such as higher antigen dose, improved adjuvants, or alternative delivery routes have been employed or proposed to improve vaccine efficacy (40). However, our results caution against reliance on virulence mechanisms defined solely in young, immunocompetent models. In immunocompromised hosts, microbial factors may have reduced, unchanged, or even increased virulence. Since elderly hosts experience higher overall morbidity despite lower virulence of Hla, we hypothesize that additional virulence determinants are responsible for the virulence of *S*. *aureus* in the immunocompromised population. These virulence determinants could include factors that promote proliferation of the pathogen (e.g., nutrient scavenging), but the hypothesis remains largely unexplored. Thus, future studies should aim at understanding the host-pathogen biology of immunocompromised hosts, with an aim to identify host-state-specific virulence factors that could be included in multivalent vaccines, thus providing protection for both the young and healthy, and the immunocompromised populations.

## Methods

### Sex as a biological variable.

Our study examined male and female animals, and similar findings are reported for both sexes.

### Mice.

C57BL/6 young mice (6–12 weeks old) were purchased from The Jackson Laboratory. C57BL/6 aged mice (16–22 months old) were either purchased directly from Jackson or purchased at 6 months of age and aged in-house. NSG mice were purchased from Jackson and used for bone marrow reconstitution. Mice were housed under specific pathogen–free conditions.

### Human volunteers.

Human neutrophils were isolated from freshly drawn peripheral blood of anonymized healthy adult donors from UCSD and the San Diego Blood Bank. Young donors were aged 18–35 years; elderly donors were aged 65–85 years. Donors were screened to exclude immunodeficiencies, ongoing infections, or medication use that could affect immune function.

### Bacterial strain and preparation.

Hla WT *S*. *aureus* strain SF8300 (USA300 lineage) and its isogenic knockout SF8300 *Δhla* were gifts from Binh Diep (UCSF, San Francisco, California, USA). To preserve phenotypic integrity, frozen glycerol stocks stored at –80°C were freshly streaked onto tryptic soy agar (TSA) or sheep blood agar plates (Hardy Diagnostics) and incubated at 37°C for 16–18 hours. Hemolytic zones were assessed to confirm Hla expression. For mouse infections and in vitro assays, bacteria were cultured in tryptic soy broth (TSB, BD Difco) at 37°C with agitation at 200 rpm. The bacteria were grown to mid-logarithmic phase (OD at 600 nm ≈ 0.7–1.0), harvested by centrifugation at 4,000*g* for 10 minutes at 4°C, and washed twice with sterile Dulbecco’s phosphate-buffered saline (DPBS; Gibco). Final bacterial suspensions were adjusted to the desired CFU concentration using OD calibration and confirmed by serial dilution and plating on TSA.

### Hla preparation and inactivation.

Purified *S*. *aureus* Hla (Sigma-Aldrich) was reconstituted in sterile DPBS at a stock concentration of 10,000 units/mL and aliquoted for single use to minimize freeze-thaw degradation. For passive vaccine preparation, Hla toxoid was generated by incubating Hla with 0.4% methanol-free formaldehyde (Thermo Fisher Scientific) at 37°C for 48 hours with gentle agitation. Complete inactivation was confirmed by loss of hemolytic activity on sheep blood agar and in vitro cytotoxicity assays. Endotoxin levels were confirmed to be less than 0.1 EU/μg protein using a Limulus Amebocyte Lysate assay (Lonza).

### Active and passive immunization.

For active immunization, mice were anesthetized briefly with isoflurane and injected subcutaneously with 1000 U of Hla toxoid in 100 μL sterile DPBS into the left flank of mice using a 30G insulin syringe on day 21 before challenge with either 1000 U of native Hla or live *S*. *aureus* (USA300 WT or *Δhla* mutant). Measurement of skin lesions and bacterial burden was conducted on day 3 and day 7 after challenge.

For passive immunization, Hla toxoid was emulsified 1:1 (v/v) with AddaVax (InvivoGen) before injection. Booster doses were given on day 14 and day 28. Serum was harvested on day 35, pooled, tested for Hla neutralizing activity, and stored at –80°C for passive immunization. A total of 1 mL of 1:2 diluted immune or control serum was administered intraperitoneally 24 hours before challenge with either native Hla (1000 U/100 μL DPBS) or live *S*. *aureus* (USA300 WT or Δ*hla* mutant, 2–3 × 10^8^ CFU/mL in 100 μL DPBS). Measurement of skin lesions and bacterial burden was conducted on day 3, 7, and 21 after challenge. All active and passive immunization studies were performed at Cedars-Sinai Medical Center.

### Skin infection model.

Subcutaneous infection was performed following an established protocol (41). Mice were anesthetized with isoflurane and inoculated subcutaneously on the back with either 100 μL of Hla (1000 U) or 2–3 × 10^8^ CFU/mL *S*. *aureus* bacterial suspension in DPBS. For the study using GI254023 (Sigma-Aldrich), 50 μL of 2 × 10^8^ CFU/mL *S*. *aureus* bacterial suspension in DPBS was used for the mouse challenge. Animals were monitored daily and skin lesions were measured using a caliper. On day 3, tissue samples (skin, kidney, spleen) were harvested aseptically, and homogenized in 0.5 mL sterile DPBS. Tissue homogenates were serially diluted, plated on TSA plates, and incubated overnight (16–18 hours) for CFU enumeration. All skin infection studies performed on young and aged mice were done at Cedars-Sinai except for the 2 studies shown in Figure 1, F–H, and Figure 3D, which were performed at UCSD.

### Bone marrow transplantation.

Donor bone marrow cells were collected from femurs and tibiae of 2-month-old (young) or 18- to 22-month-old (aged) C57BL/6 mice. Cells were flushed with sterile DPBS containing 2% FBS and filtered through a 70 μm strainer. Red blood cells were lysed with lysis buffer, and nucleated cells were counted using trypan blue exclusion. A total of 1 × 10^5^ viable bone marrow cells were injected intrahepatically into 1- to 3-day-old NSG pups (The Jackson Laboratory) using a 30G needle under sterile conditions. Mice were weaned at 3 weeks of age and monitored for 16 weeks. Reconstitution of myeloid lineages was confirmed by flow cytometry for CD45^+^ and CD11b^+^ cells in spleen and peripheral blood. The bone marrow transplantation study was performed at Cedars-Sinai Medical Center.

### Cyclophosphamide-induced leukopenia in mice.

Cyclophosphamide monohydrate (Sigma-Aldrich) was dissolved in sterile DPBS. Mice were administered 3 doses of cyclophosphamide (75 mg/kg body weight) every 48 hours via intraperitoneal injection. DPBS without cyclophosphamide monohydrate was used as a negative control. One day after the third drug injection, the mice were subcutaneously challenged with *S*. *aureus*. All infections using the leukopenia mouse model were performed at UCSD.

### Neutrophil and macrophage isolation.

Murine bone marrow neutrophils were isolated using a MojoSort Mouse Neutrophil Isolation Kit (BioLegend) following the manufacturer’s protocol. Human neutrophils were isolated from 30 mL whole-blood samples from volunteers at UCSD and the San Diego Blood Bank. Human neutrophils were isolated and purified using 1-Step Polymorphs (Fresenius Kabi Norge AS), following the manufacturer’s instructions. Cells were resuspended in RPMI 1640 (Gibco) containing 10% FBS.

BMDMs were generated from young (6–12 weeks) or aged (18–22 months) C57BL/6 mice. Bone marrow cells (1 × 10^7^) were cultured overnight in DMEM supplemented with 10% FCS and recombinant M-CSF in tissue culture–treated dishes. Afterwards, non-adherent cells were transferred to non–tissue culture–treated Petri dishes to eliminate adherent stromal cells and differentiated for an additional 4–5 days at 37°C. Mature macrophages were detached using EDTA dissociation solution, counted, and reinoculated at 5 × 10^4^ cells per well in 96-well plates in DMEM containing 10% FCS and M-CSF, and incubated overnight prior to experimentation.

### Cytotoxicity assay.

Neutrophils were plated in 96-well flat-bottom tissue culture plates (Corning) at 5 × 10^6^ cells per well in 100 μL Hanks’ balanced salt solution (HBSS without Ca^2+^ and Mg^2+^, Invitrogen) with 0.5% BSA. Cells were incubated with 10 U/mL native Hla or vehicle control at 37°C with 5% CO_2_ for 3 hours. Cell lysis was quantified by measuring lactate dehydrogenase (LDH) release using a colorimetric LDH Cytotoxicity Assay Kit (Takara Clontech) according to the manufacturer’s instructions. Absorbance was read at 490 nm using a SmartReader 96 (Accuris) microplate reader. Cytotoxicity was calculated as the percentage of maximum lysis.

### Determination of cytokines in neutrophil and macrophage culture supernatants after challenge with Hla.

Enzyme-linked immunosorbent assay (ELISA) was performed with an IL-1β ELISA MAX Deluxe Set (BioLegend) using supernatants from Hla-treated mouse and human neutrophils. Neutrophils purified from mouse bone marrow or human blood samples were incubated with LPS (200 ng/mL) (eBioscience) for 4 hours, then treated with Hla (10 U/mL) for 2 hours at 37°C. The supernatants were then tested for IL-1β levels following the manufacturer’s instructions. Absorbance was measured at 450 nm using a SmartReader 96. Cytokine concentrations were determined by interpolation from a standard curve generated with known concentrations of recombinant IL-1β.

For the determination of cytokines after challenge of BMDMs with Hla, BMDMs were stimulated with 0.1 ng/mL LPS or 10 ng/mL Pam3CSK4 (InvivoGen) for 3 hours, then treated with 10 U/mL Hla for another 3 hours. IL-1β, TNFα, and MIP-2 in culture supernatants were detected using TNFα and MIP-2 ELISA MAX Deluxe Set (BioLegend). For the detection of IL-1β, BMDMs were also stimulated with heat-killed *S*. *aureus* Δ*hla* (1 × 10^8^ CFU) plus Hla (250 U/mL) for 6 hours.

### qPCR.

Total RNA was isolated from tissue or neutrophil lysates using the RNeasy Mini Kit (Qiagen), treated with TURBO DNA MiniPrep kit (Invitrogen) for DNA removal, and reverse transcribed with SuperScript III (Invitrogen). qPCR was performed using SYBR Green Master Mix (Bio-Rad) on a CFX96 Touch Real-Time PCR Detection System (Bio-Rad). Relative mRNA levels were normalized to mouse 18s or GAPDH and calculated using the 2^–ΔCt^ method. Primer (Integrated DNA Technologies) sequences for ADAM10 and housekeeping genes are as follows. For mouse: *Adam10* Forward, 5′-TCATGGTGAAACGCATAAGAATCA -3′ and *Adam10* Reverse, 5′-CCAGACCAAGTACGCCATCA-3′; *18s* Forward, 5′-GCCGCTAGAGGTGAAATTCTT-3′ and *18s* Reverse, 5′-CGTCTTCGAACCTCCGACT-3′. For human: *ADAM10* Forward, 5′-CTGGCCAACCTATTTGTGGAA-3′ and *ADAM10* Reverse, 5′-GACCTTGACTTGGACTGCACTG-3′; *GAPDH* Forward, 5′-GAAGGGCTCATGACCACAGTCCAT-3′ and *GAPDH* Reverse, 5′-TCATTGTCGTACCAGGAAATGAGCTT-3′.

### ADAM10 activity assay.

ADAM10 enzymatic activity was measured using the ADAM10 Fluorogenic Assay Kit (BPS Biosciences). Neutrophil lysates were prepared by lysing cells in RIPA lysis buffer (MilliporeSigma) with complete protease inhibitor cocktail (Sigma-Aldrich) on ice for 20 minutes, followed by a high-speed centrifugation at 14,500*g* for 10 minutes at 4°C. Neutrophil lysates or tissue homogenates (5 μg protein per reaction) were used for the reaction in each well of 96-well plates with or without the ADAM10-specific inhibitor GI254023. Fluorescence (Ex/Em: 485/538 nm) was recorded after a 5-minute reaction at room temperature using a fluorescence microplate reader.

### Flow cytometric analysis of neutrophil ADAM10 surface expression.

Neutrophils, with or without Hla pretreatment, were washed with FACS buffer (DPBS + 1% BSA), blocked with anti-CD16/CD32 Fc block (catalog 14-0161-82, Invitrogen) or Fc receptor binding inhibitor (catalog 14-9161-73, Invitrogen), and then stained with fluorophore-conjugated monoclonal antibodies against CD11b (catalog 101227, BioLegend), Ly6G (catalog 127605, BioLegend), or CD15 (catalog 301904, BioLegend), and ADAM10 (catalog IC1427P, BioLegend) to detect surface ADAM10 on neutrophils. UltraComp eBeads Compensation beads (Invitrogen) were used for single fluorescence control. Flow cytometry was performed on a BD FACSCanto II flow cytometer (BD Biosciences) and analyzed using FlowJo_v10.9.0_CL (flowjo.com).

### Hla binding assay.

Hla binding to neutrophils was assessed using Hla toxoid labeled with Alexa Fluor 647, using Alexa Fluor 647 protein labeling kit (Invitrogen). After co-incubation with the fluorophore-labeled Hla toxoid at 37°C for 15 minutes, the cells were washed twice and stained with fluorophore-conjugated monoclonal antibodies against CD11b (catalog 101207, BioLegend) for confirmation of isolated neutrophils. Flow cytometry was performed on a BD FACSCanto II flow cytometer and analyzed using FlowJo_v10.9.0_CL.

### Statistics.

The sample size, statistical methods, and relevant details are provided in the figure legends and the main text. GraphPad Prism 10.4.1 was used for all statistical analyses. Two-group analyses used unpaired, 2-tailed Student’s *t* test or non-parametric Mann-Whitney *U* test. Comparisons of multiple groups used 1-way ANOVA with the Kruskal-Wallis test for non-normality. Data are presented as mean ± SEM unless otherwise indicated.

### Study approval.

All animal studies were reviewed and approved by the Institutional Animal Care and Use Committee (IACUC) at the UCSD and Cedars-Sinai Medical Center (Los Angeles, California, USA), and were conducted in accordance with institutional guidelines and the NIH *Guide for the Care and Use of Laboratory Animals* (National Academies Press, 2011).

Studies involving human participants were reviewed and approved by the Institutional Review Board (IRB) of UCSD. Written informed consent was obtained from all human volunteers prior to participation. Blood samples from the San Diego Blood Bank (San Diego, California, USA) were collected from deidentified donors under protocols approved by the respective institutional review bodies, and no additional consent was required for anonymized sample use.

### Data availability.

All data supporting the findings of this study are available within the paper, the supplemental material, and in the Supporting Data Values file.

## Author contributions

XD and CWT performed most of the experiments. XD, CWT, and GYL designed the study. EB, HG, and JS participated in collecting the human samples and the in vitro experiments. CMT, IAH, CG, and BL participated in the mouse assays and infection experiments. XD, CWT, and GYL analyzed the data. XD, CWT, VN, and GYL wrote the manuscript, with all authors providing input. The order of co–first authors was determined by mutual agreement among co–first authors.

## Conflict of interest

The authors have declared that no conflict of interest exists.

## Funding support

This work is the result of NIH funding, in whole or in part, and is subject to the NIH Public Access Policy. Through acceptance of this federal funding, the NIH has been given a right to make the work publicly available in PubMed Central.

- NIH grants R01AI179098, R01AI144694, and R01AI181321 (all to GYL).

## Supplementary Material

Supplemental data

Supporting data values

## Version 1 06/08/2026

Electronic publication
