# Supplementary material for: Reduced efficacy of an anti-toxin vaccine from senescence-driven attenuation of toxin virulence
Source: JCI Insight. 2026 Jun 8;11(11):e199834. doi: 10.1172/jci.insight.199834 (PMC13313543; doi:10.1172/jci.insight.199834)
Supplement: Supplemental data [file jciinsight-11-199834-s141.pdf]

SUPPLEMENTAL MATERIAL

Reduced efficacy of an anti-toxin vaccine from senescence-driven attenuation of toxin virulence

Authors: Xin Du[1,2§], Ching-Wen Tseng[3,|| §*], Elisabet Bjånes[1,4], Hunter Gage[1,4], Jaclyn Swan[5], Chih-Ming Tsai[1,2], Irshad Hajam[1,2], Cesia Gonzalez[1,2], Brian Lin[1,2], Victor Nizet[1,4], George Y Liu[1,2*]

Affiliations:

[1]Department of Pediatrics, University of California San Diego, San Diego, California, United States.

[2]Division of Infectious Diseases, Rady Children's Hospital San Diego, San Diego, California, United States.

[3]Orca Bio, Menlo Park, California, United States.

[4]Department of Pharmacy, University of California San Diego, San Diego, California, United States.

[5]Department of Ecological Plant and Animal Sciences, La Trobe University, Melbourne, Australia.

[§]Equal contributions

[||]Former address: Department of Pediatric, Cedars-Sinai Medical Center, Los Angeles, California, United States.

*Corresponding Author Email: gyliu@ucsd.edu; dolorsas@gmail.com

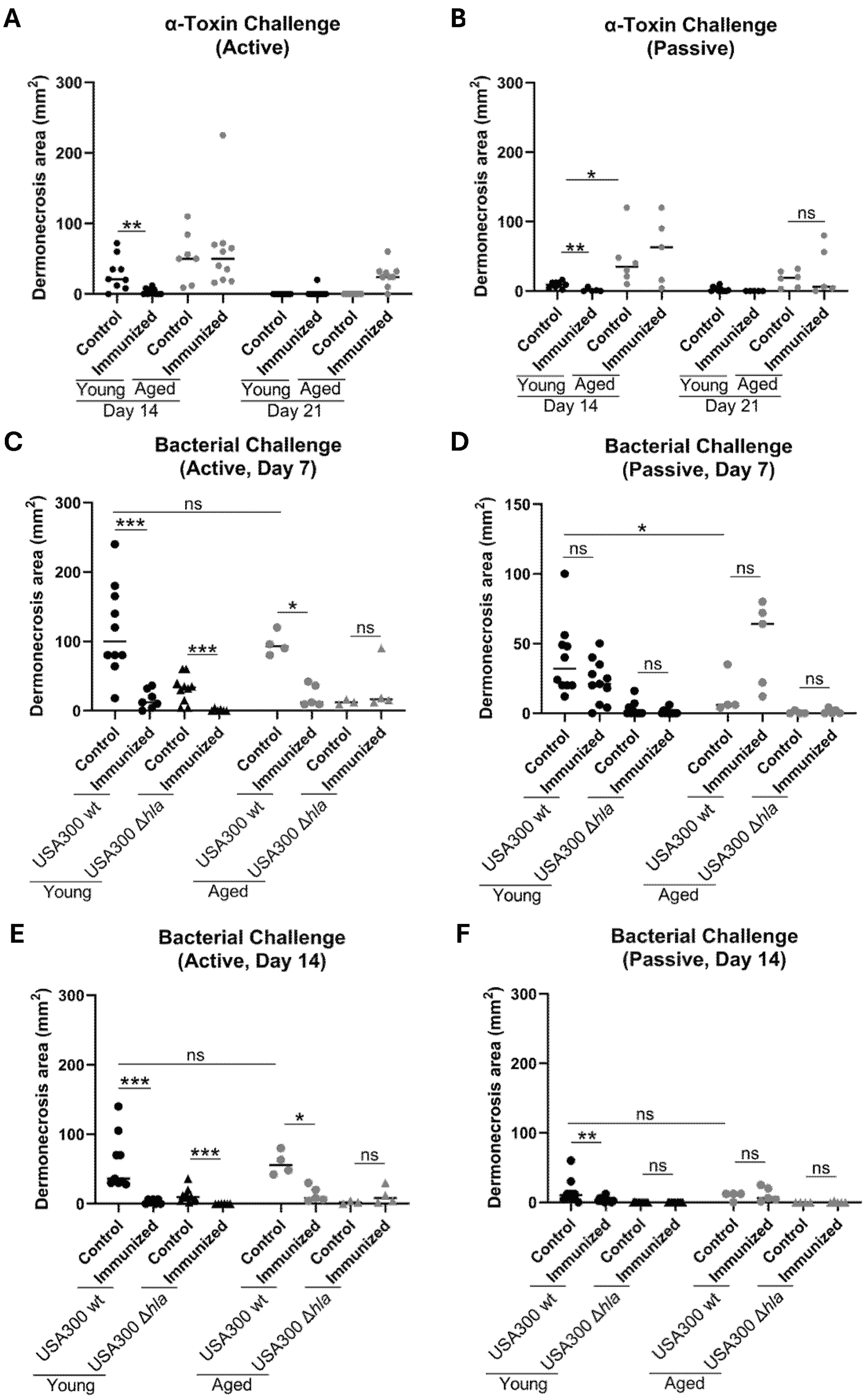
A
α-Toxin Challenge (Active)
B
α-Toxin Challenge (Passive)
C
Bacterial Challenge (Active, Day 7)
D
Bacterial Challenge (Passive, Day 7)
E
Bacterial Challenge (Active, Day 14)
F
Bacterial Challenge (Passive, Day 14)
Dermonecrosis area (mm²)
Control
Immunized
Young
Aged
Day 14
Day 21
USA300 wt
USA300 Δhla
ns
*
**
***

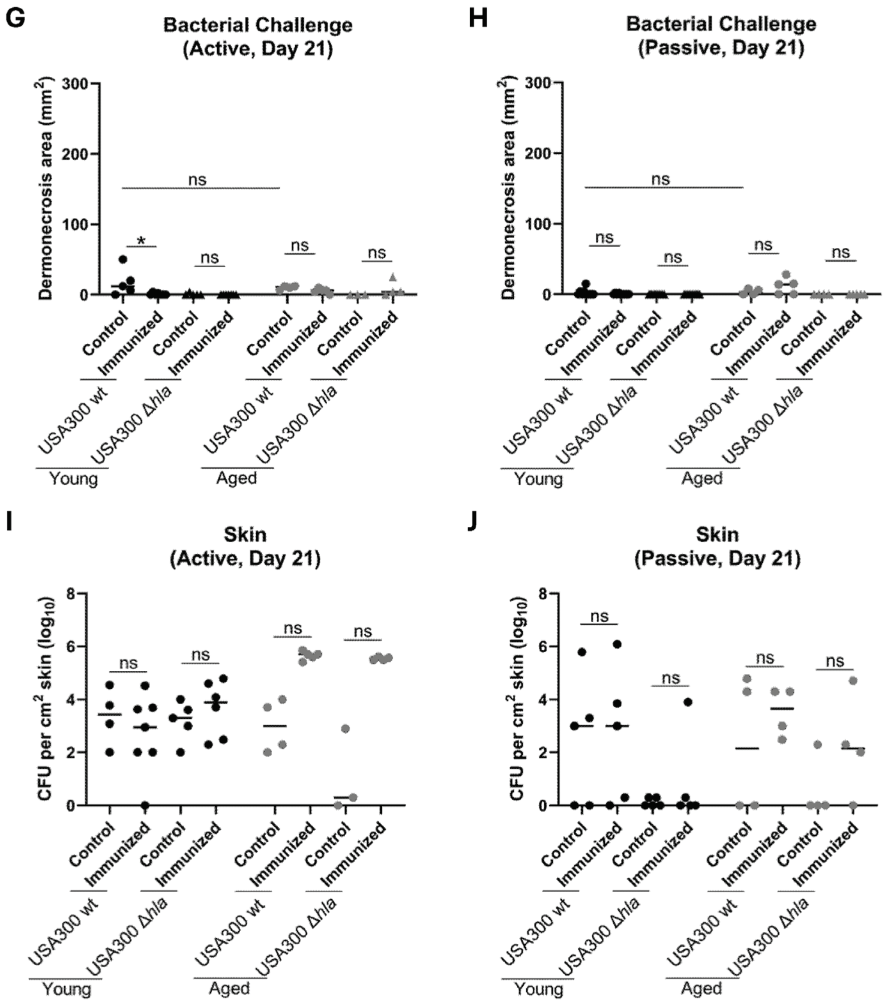

Fig. S1. Quantitation of bacterial burden and skin lesions following anti-Hla immunization and Hla or *S. aureus* challenge of young and aged mice. Young and aged mice were administered active or passive anti-Hla immunization then challenged subcutaneously with Hla or *S. aureus*. (**A-B**) Active and passive immunization - Skin lesion sizes 14 and 21 days after Hla challenge (n=5-9 mice). (**C-D**) Active and passive immunization - Skin lesion sizes 7 days after *S. aureus* infection (n=9-11 young, n=4-7 aged). (**E-F**) Active and passive immunization - Skin lesion sizes 14 days after *S. aureus* infection (n=9-11 young, n=4-7 aged). (**G-H**) Active and passive immunization - Skin lesion sizes 21 days after *S. aureus* infection (n=9-11 young, n=4-7 aged). (**I-J**) Active and passive immunization - Bacterial burden 21 days post-infection (n=4-6). Each data point represents an individual mouse. *$p < 0.05$, **$p < 0.01$, and ***$p < 0.001$; ns, not significant. Line represents mean. Kruskal-Wallis non-parametric one-way ANOVA test (**A-J**).

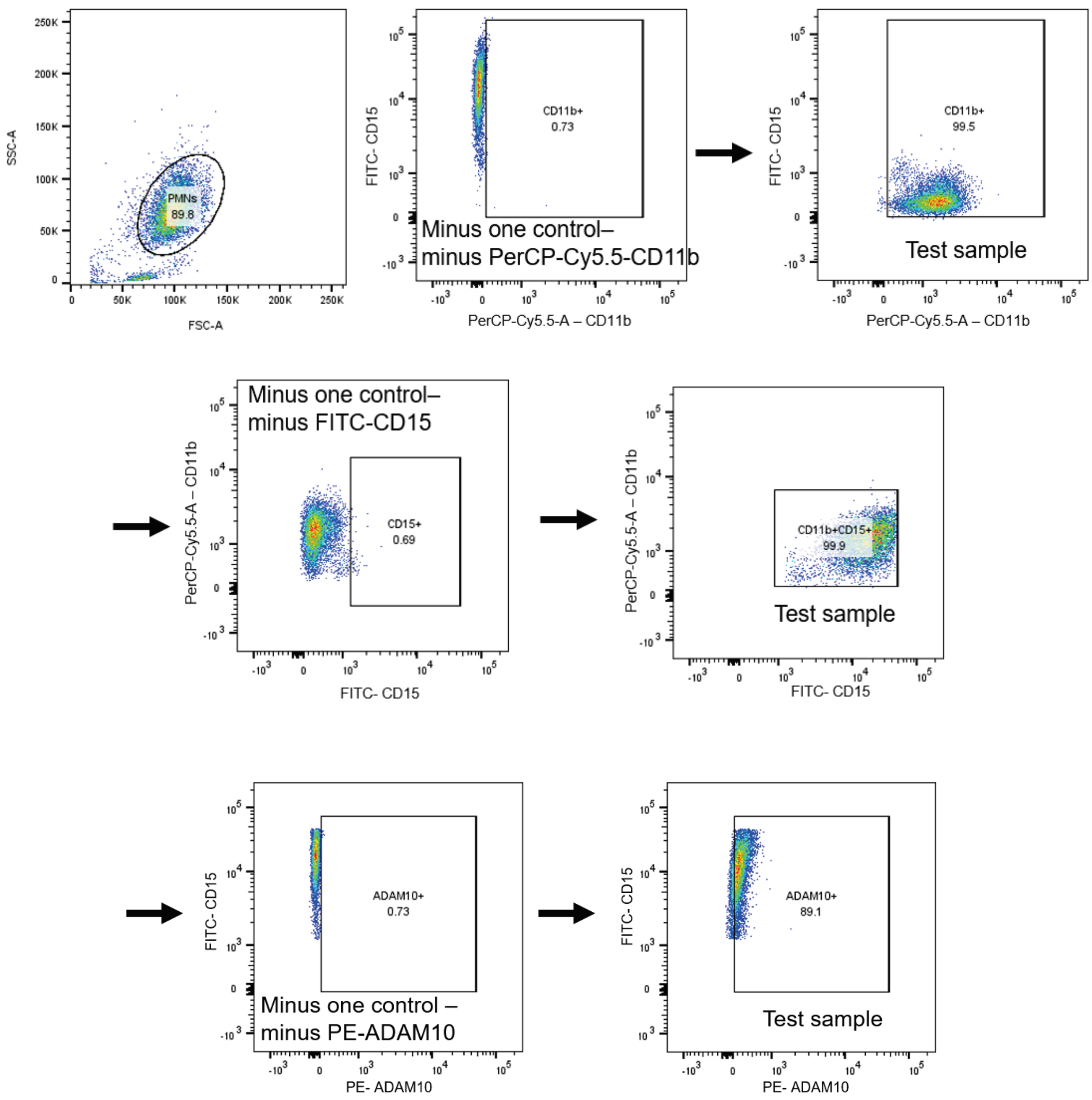

Fig. S2. Flow Cytometer gating strategy for the assessment of ADAM10 expression on PMNs. Neutrophils purified from young and aged healthy human volunteers were untreated or Hla pretreated for 30 minutes and stained with both fluorescence-conjugated antibodies against CD markers for neutrophils and ADAM10.

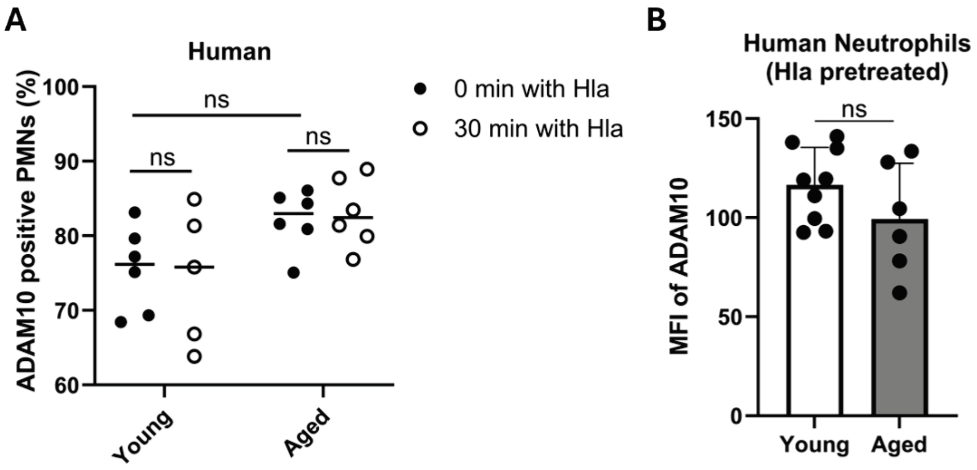

Fig. S3. ADAM10 expression by human neutrophils following Hla treatment. (**A**) Percentage of ADAM10-positive neutrophils 30 minutes after Hla treatment (n=5-6). (**B**) MFI of ADAM10 expression 30 minutes after Hla treatment (n=6).Line represents mean (**A**); the data is presented as mean ± SEM of biological replicates (**B**). ns, not significant, Kruskal-Wallis non-parametric one-way ANOVA test (**A**); two-tailed non-parametric Mann-Whitney U test (**B**).

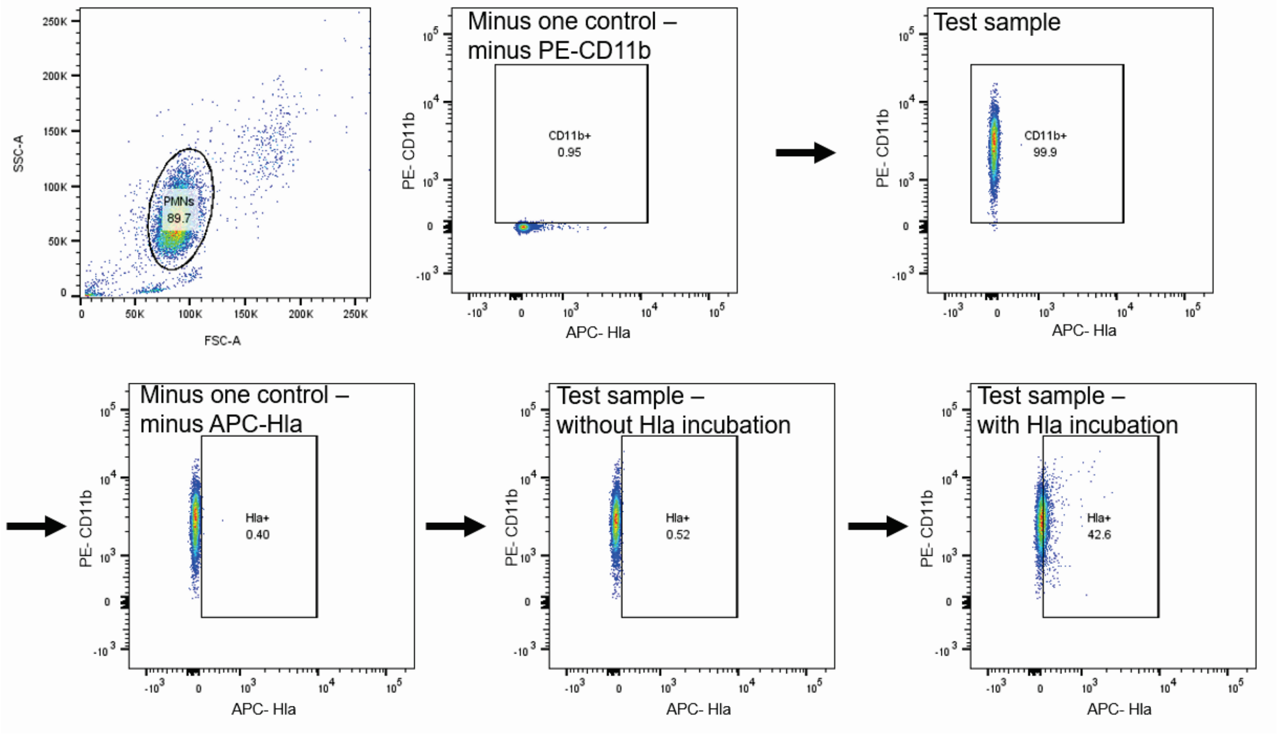

Fig. S4. Flow Cytometer Gating Strategy for the assessment of Hla binding to PMNs.

Neutrophils purified from young and aged human volunteers were untreated or pretreated with fluorescence-conjugated Hla for 15 minutes, then stained with fluorescence-conjugated antibody against the CD marker for neutrophils.

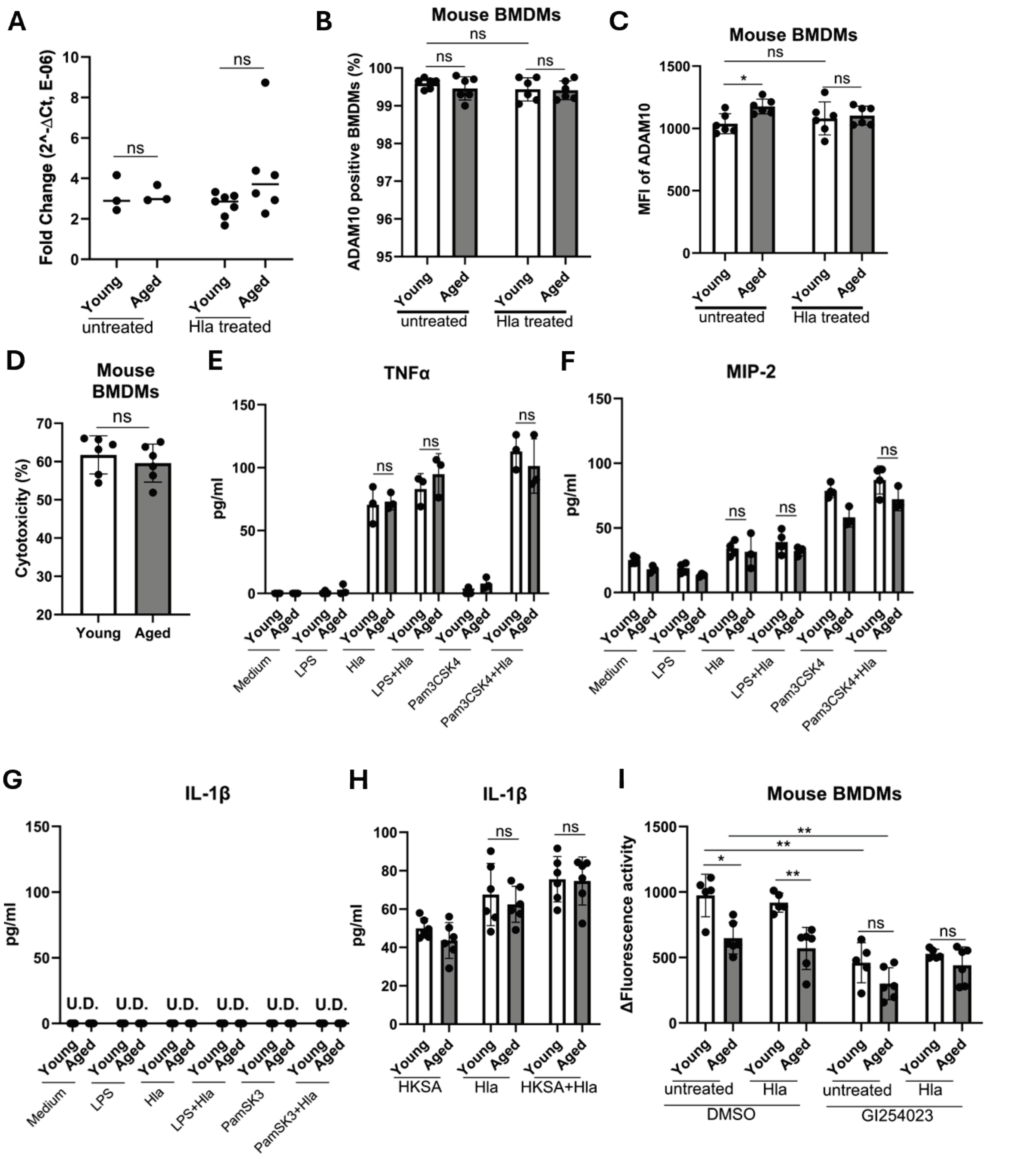

Fig. S5. ADAM10 expression and enzymatic activity, and the proinflammatory effect of Hla on bone marrow-derived macrophages (BMDMs) from young and aged mice. (**A**) ADAM10 mRNA expression in BMDMs from young and aged mice, treated with Hla, quantified by qPCR, and normalized to housekeeping gene expression. (**B-C**) ADAM10 expression measured with Flow Cytometry. Percentage of ADAM10-positive BMDMs (**B**) and mean

fluorescence intensity (MFI) of ADAM10 expression (**C**) assessed by flow cytometry in untreated or Hla-treated BMDMs from young and aged mice. (**D**) Hla (10 U/ml) lysis of BMDMs derived from young and aged mice assessed using LDH assay. Percent cell lysis in untreated controls – young 7.7, aged 7.6 (n=6 mice). (**E–G**) Cytokine production following Hla (10 U/ml) treatment measured by ELISA. TNF-α (**E**), MIP-2 (**F**), and (**G**) IL-1β production by BMDMs from young and aged mice following stimulation with medium, LPS, Hla (10 U/ml), or LPS plus Hla (10 U/ml), Pam3CSK4, or Pam3CSK4 plus Hla (10 U/ml). (**H**) IL-1β production by BMDMs from young and aged mice following stimulation with heat-killed SA Δ*hla* (HKSA), Hla (250 U/ml), or HKSA plus Hla (250 U/ml). (**I**) Enzymatic activity of ADAM10 in lysates of BMDM from young and aged mice, measured using a fluorogenic substrate. The BMDMs were treated with DMSO vehicle or the ADAM10-specific inhibitor GI254023 dissolved in DMSO, with or without Hla (10 U/ml). Each data point represents an individual biological replicate. Data are presented as mean ± SEM. Two-tailed non-parametric Mann-Whitney U test (**D-H**). Kruskal-Wallis non-parametric one-way ANOVA test (**A-C, I**). *$p < 0.05$, **$p < 0.01$; ns, not significant.

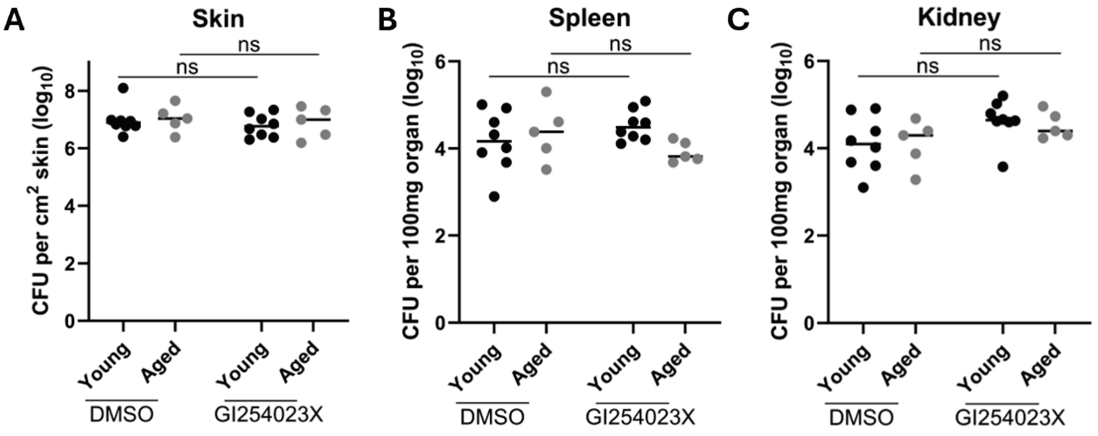

Fig. S6. Bacterial burden in young and aged mice treated with ADAM10 inhibitor and challenged with *S. aureus*. Young and aged mice were treated with DMSO carrier or ADAM10-specific inhibitor GI254023X, then challenged with WT *S. aureus*. Shown are bacterial burden from the skin (**A**), spleen (**B**), and Kidneys (**C**), 3 days post-infection. Line represents mean. Kruskal-Wallis non-parametric one-way ANOVA test (**A-C**). *S. aureus*: USA300 WT SF8300.

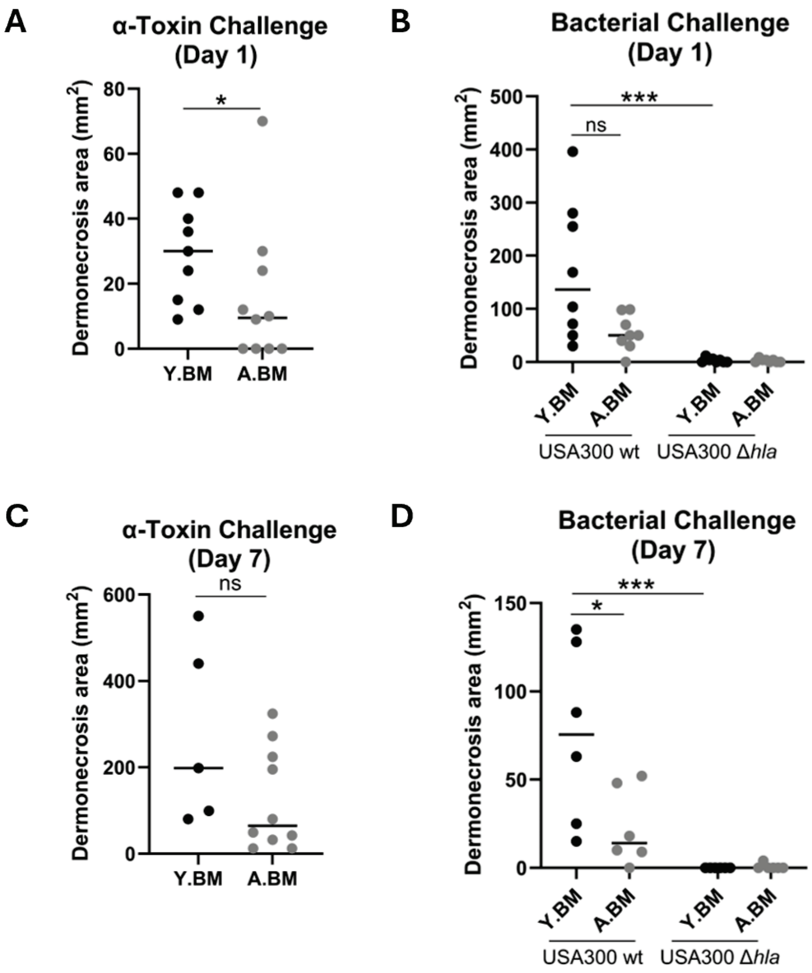

Fig. S7. Quantitation of skin lesions after Hla or *S. aureus* challenge in recipients of young and aged bone marrow. NSG mice received bone marrow from young or aged mice. At 16 weeks of age, the mice were challenged with Hla, WT or Δ*hla S. aureus*. (**A-B**) Skin lesion sizes 1 and 7 days after Hla challenge. (**C-D**) Skin lesion sizes 1 and 7 days after *S. aureus* challenge. Line represents mean. *p < 0.05, **p < 0.01, and ***p < 0.001; ns, not significant. Two-tailed non-parametric Mann-Whitney U test (**A-D**). Y.BM: NSG recipients of bone marrow from young C57BL/6 mice; A.BM: NSG recipients of bone marrow from aged C57BL/6 mice.
